# Cerebrospinal Fluid Biomarkers in Autopsy-Confirmed Alzheimer Disease and Frontotemporal Lobar Degeneration

**DOI:** 10.1212/WNL.0000000000200040

**Published:** 2022-03-15

**Authors:** Niklas Mattsson-Carlgren, Lea T. Grinberg, Adam Boxer, Rik Ossenkoppele, Magnus Jonsson, William Seeley, Alexander Ehrenberg, Salvatore Spina, Shorena Janelidze, Julio Rojas-Martinex, Howard Rosen, Renaud La Joie, Orit Lesman-Segev, Leonardo Iaccarino, Gwendlyn Kollmorgen, Peter Ljubenkov, Udo Eichenlaub, Maria Luisa Gorno-Tempini, Bruce Miller, Oskar Hansson, Gil Dan Rabinovici

**Affiliations:** From the Clinical Memory Research Unit (N.M.-C., R.O., S.J., O.H.), Faculty of Medicine, Department of Neurology (N.M.-C.), Skåne University Hospital, and Wallenberg Center for Molecular Medicine (N.M.-C.), Lund University, Sweden; Department of Neurology (L.T.G., A.B., W.S., A.E., S.S., J.R.-M., H.R., R L.J., O.L.-S., L.I., P.L., M.L.G.-T., B.M., G.D.R.), Memory and Aging Center, Department of Pathology (L.T.G., W.S.), and Department of Radiology and Biomedical Imaging (G.D.R.), University of California San Francisco; Alzheimer Center Amsterdam (R.O.), Department of Neurology, Amsterdam Neuroscience, Vrije Universiteit Amsterdam, Amsterdam UMC, the Netherlands; Department of Clinical Chemistry (M.J.), Skåne University Hospital, Malmö, Sweden; Department of Integrative Biology (A.E.), University of California, Berkeley; Roche Diagnostics GmbH (G.K., U.E.), Penzberg, Germany; and Memory Clinic (O.H.), Skåne University Hospital, Malmö, Sweden.

## Abstract

**Background and Objectives:**

To determine how fully automated Elecsys CSF immunoassays for β-amyloid (Aβ) and tau biomarkers and an ultrasensitive Simoa assay for neurofilament light chain (NFL) correlate with neuropathologic changes of Alzheimer disease (AD) and frontotemporal lobar degeneration (FTLD).

**Methods:**

We studied 101 patients with antemortem CSF and neuropathology data. CSF samples were collected a mean of 2.9 years before death (range 0.2–7.5 years). CSF was analyzed for Aβ_40_, Aβ_42_, total tau (T-tau), tau phosphorylated at amino acid residue 181 (P-tau), P-tau/Aβ_42_ and Aβ_42_/Aβ_40_ ratios, and NFL. Neuropathology measures included Thal phases, Braak stages, Consortium to Establish a Registry for Alzheimer's Disease (CERAD) scores, AD neuropathologic change (ADNC), and primary and contributory pathologic diagnoses. Associations between CSF biomarkers and neuropathologic features were tested in regression models adjusted for age, sex, and time from sampling to death.

**Results:**

CSF biomarkers were associated with neuropathologic measures of Aβ (Thal, CERAD score), tau (Braak stage), and overall ADNC. The CSF P-tau/Aβ_42_ and Aβ_42_/Aβ_40_ ratios had high sensitivity, specificity, and overall diagnostic performance for intermediate-high ADNC (area under the curve range 0.95–0.96). Distinct biomarker patterns were seen in different FTLD subtypes, with increased NFL and reduced P-tau/T-tau in FTLD–TAR DNA-binding protein 43 and reduced T-tau in progressive supranuclear palsy compared to other FTLD variants.

**Discussion:**

CSF biomarkers, including P-tau, T-tau, Aβ_42_, Aβ_40_, and NFL, support in vivo identification of AD neuropathology and correlate with FTLD neuropathology.

**Classification of Evidence:**

This study provides Class II evidence that distinct CSF biomarker patterns, including for P-tau, T-tau, Aβ_42_, Aβ_40_, and NFL, are associated with AD and FTLD neuropathology.

Alzheimer disease (AD) and frontotemporal lobar degeneration (FTLD) are common causes of dementia. AD is characterized by β-amyloid (Aβ) and tau aggregation,^1^ while FTLD is most often associated with tau (FTLD-tau) or TAR DNA-binding protein 43 (TDP-43) (FTLD-TDP) aggregation. Clinical presentations have varying correlations with neuropathology in AD and FTLD.^2,3^ Biomarkers are therefore needed to assist in diagnosis and to accelerate drug development.

PET and CSF biomarkers are available for neurodegenerative diseases.^4^ CSF biomarkers include Aβ_42_ and Aβ_40_, total tau (T-tau), phosphorylated tau (P-tau), and neurofilament light chain (NFL). PET for Aβ and tau has been validated with neuropathology,^5,6^ but few studies have reported such validation for CSF AD biomarkers and mainly used older assays.^7,8^ CSF studies in FTLD focused on differentiation vs AD^9-12^ or differences between FTLD variants.^13-17^ Postmortem FTLD studies are rare, with some but not all^15^ finding reduced P-tau^14,16^ or P-tau/T-tau^17,18^ in FTLD-TDP compared to FTLD-tau.

Older CSF assays were hampered by variability, but this has been overcome with novel, fully automated assays.^19^ We tested associations between Elecsys Aβ_42_, Aβ_40_, T-tau and P-tau, and NFL (using Simoa) assays and neuropathology. Our primary research question was whether distinct CSF biomarker patterns, including for P-tau, T-tau, Aβ_42_, Aβ_40_, and NFL, were associated with AD and FTLD neuropathology. We hypothesized that AD neuropathologic changes (ADNC) would be associated with reduced Aβ_42_/Aβ_40_ and increased P-tau/Aβ_42_ also when coexisting with other pathologies. We also expected to find reduced tau markers in progressive supranuclear palsy (PSP; a variant of FTLD-tau)^13,20^ and lower P-tau/T-tau in FTLD-TDP.^14,16,17^

## Methods

### Study Group

Patients were recruited from the University of California San Francisco (UCSF) Memory and Aging Center's Alzheimer's Disease Research Center. They met diagnostic criteria for different FTLD syndromes, including behavioral variant frontotemporal dementia (bvFTD),^21^ corticobasal syndrome (CBS),^22^ nonfluent variant primary progressive aphasia,^23^ PSP,^24^ semantic variant primary progressive aphasia,^23^ or frontotemporal dementia (FTD)–amyotrophic lateral sclerosis,^25^ or had probable AD-type dementia.^26^ Five patients did not meet any research diagnostic criteria (but had syndromes considered most compatible with AD, bvFTD, or PSP). All participants underwent a medical history and physical examination, a structured caregiver interview, lumbar puncture (LP), and neuropsychological testing. We did not have a criterion for maximum time difference between LP and death (mean time 2.9 years [SD 1.8 years, range 0.2–7.5 years]). We included all eligible study participants.

### Standard Protocol Approvals, Registrations, and Patient Consents

Written informed consent was obtained from all participants or their assigned surrogate decision makers. The UCSF institutional review board for human research approved the study.

### CSF Biomarkers and Cognitive Testing

CSF was obtained following protocols from Alzheimer's Disease Neuroimaging Initiative.^27^ In short, CSF was sampled in the morning after an overnight fast with a 20- or 24-gauge spinal needle. Samples were transferred from collection tubes into polypropylene tubes and frozen within 1 hour of sampling. Samples were shipped frozen to Lund University and Skåne University Hospital, where they were analyzed for Elecsys (Roche Diagnostics International Ltd, Rotkreuz, Switzerland) CSF AD biomarkers^19^ and NFL was analyzed by a Simoa method (NF-light Simoa Assay Advantage Kit; Quanterix Inc, Billerica, MA,). Aβ_42_ and Aβ_40_ have been associated with Aβ pathology; T-tau has been associated with axonal injury; P-tau has been associated with tau pathology in AD; and NFL has been associated with injury preferentially in large-diameter myelinated axons.^28^ Cognitive testing with Mini-Mental State Examination (testing overall cognitive status^29^), Trail Making Test Part B (testing speed of processing and executive function^30^), and California Verbal Learning Test-II (an episodic memory test^31^) was done at a median of 2 (interquartile range 1–6) days before LP.

### Neuropathology

Comprehensive neuropathologic assessments were performed by investigators at the UCSF Neurodegenerative Disease Brain Bank who were blinded to CSF results, following previously described procedures.^32^ Classifications of AD,^33^ FTLD-TDP, and FTLD-tau (including PSP and corticobasal degeneration [CBD]) followed standard neuropathologic criteria.^33-35^ For each patient, a main (primary) pathology was defined as the pathology that was most likely to explain the clinical syndrome on the basis of its anatomic location and degree of pathologic change. For each patient, other (contributing) pathologies were also defined as the pathologic changes that could explain some of the symptoms in addition to the primary pathology. We also determined the AD Thal amyloid phase,^36^ indicating topographic extent of Aβ plaque pathology; Braak neurofibrillary tangle stage,^37^ indicating the topographic extent of tau neurofibrillary pathology; and Consortium to Establish a Registry for Alzheimer's Disease (CERAD) score,^38^ indicating the density of neocortical neuritic plaques. Thal phase, Braak stage, and CERAD score were aggregated in the ADNC score.^32^ ADNC has 4 levels: none, low, intermediate, and high. One of our main aims was to test associations between CSF biomarkers and AD pathology. To avoid bias from subjective interpretations, we used intermediate to high ADNC as an indicator of AD pathology^39^ independently of whether the neuropathologists had identified AD as a primary or contributory pathology to the patient's clinical syndrome (4 patients who presented clinically with PSP, CBS, or bvFTD had intermediate ADNC, although AD changes were not considered primary or contributing).

### Statistics

Linear regression models adjusted for age, sex, and time between LP and death were used to test associations between biomarkers (as outcomes) and neuropathologic features (as categorical predictors). The overall effect of each feature (across all levels, e.g., Thal phases 0–5) on biomarkers was tested by likelihood ratio tests comparing nested models with and without neuropathologic score as a predictor. Associations between dichotomous ADNC (none-low vs intermediate-high) and dichotomous biomarker status were tested with the Fisher exact test. Receiver operating characteristics (ROC) analyses were used to test diagnostic performance (by area under the ROC curve, AUC) of different biomarkers for dichotomous ADNC. This used predictions from logistic regression models with ADNC as the outcome and biomarkers as predictors, with CIs generated by a bootstrap procedure, using the pROC package.^40^ An internal 10-fold cross-validation of AUC estimates and CIs was done using influence curves with the cvAUC package.^41^ Sensitivity and specificity were tested using a priori cut points. When indicated, we used Akaike information criterion (AIC) for model selection (a difference of >2 favors the model with smaller AIC). Sensitivity analyses were done on subsets of participants. Values of *p* were considered significant at *p* < 0.05, 2 tailed. When mentioned, multiplicity correction was done with the Bonferroni method. Statistical analyses were done in R (version 4.0.2, R Foundation for Statistical Computing, Vienna, Austria).

### Data Availability

Per the UCSF Memory and Aging Center data-sharing policy, data can be made available on reasonable request via our online portal.^42^

## Results

Group characteristics by primary neuropathologic diagnoses (AD, FTLD-tau [CBD], FTLD-tau [PSP], FTLD-TDP, and others) are shown in Table 1. Frequencies of copathologies are shown in Table 1 (by primary neuropathology) and eTable 1, links.lww.com/WNL/B777 (by clinical diagnosis). Only 3 of 15 patients with a clinical AD diagnosis had a non-AD primary pathologic diagnosis, and 2 of these 3 had AD as a contributing diagnosis (eTable 1). Conversely, AD was the primary underlying pathology in only 4 of 85 with a non-AD diagnosis. Seventy-five patients had a clinical diagnosis of an FTD syndrome (PSP–Richardson syndrome, CBS, bvFTD, semantic variant primary progressive aphasia, nonfluent variant primary progressive aphasia, or amyotrophic lateral sclerosis [most often together with bvFTD]), and all but 3 of these had an FTLD neuropathologic variant as the primary pathologic diagnosis. Most heterogeneity in underlying primary neuropathology was seen in CBS; 10 patients had FTLD-tau (CBD), 8 had FTLD-tau (PSP), 1 had FTLD-tau (Pick disease), 1 had FTLD-TDP, and 1 lacked evidence of neurodegeneration. eFigure 1 shows CSF biomarkers by clinical diagnosis.

**Table 1:**
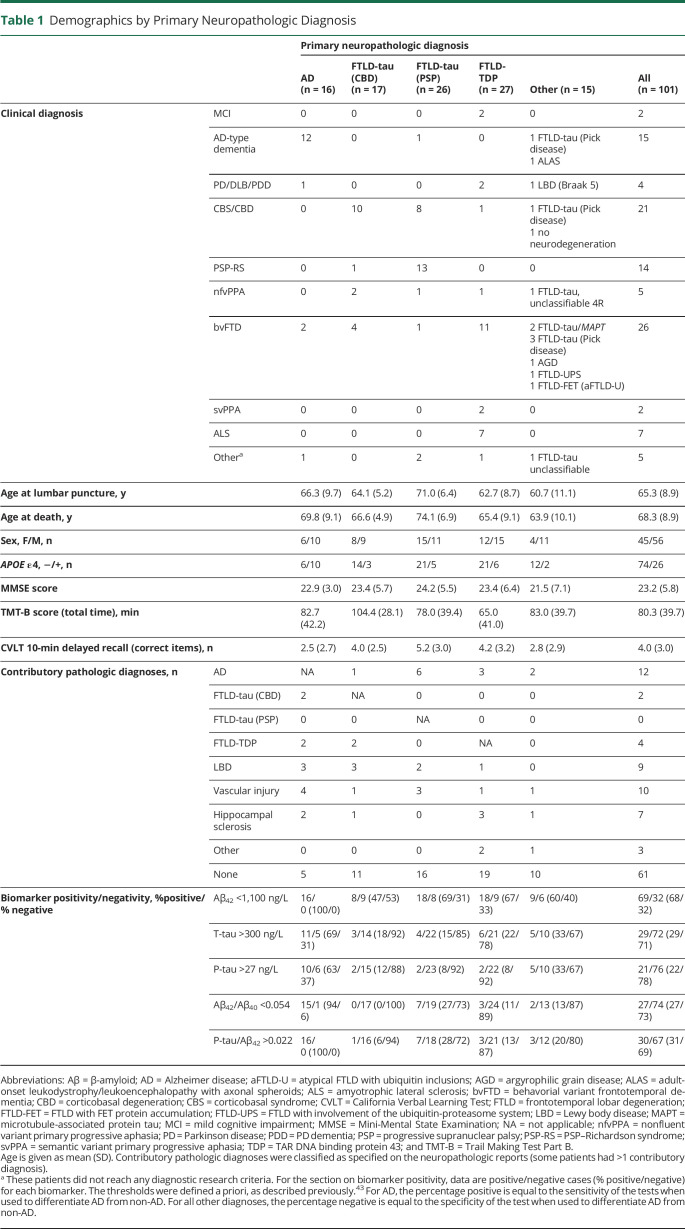
Demographics by Primary Neuropathologic Diagnosis

Frequencies of AD-related neuropathologic scores, including Thal phase, Braak stage, CERAD score, and ADNC, are shown in eFigure 2, links.lww.com/WNL/B777. Most patients had either CERAD scores of none or frequent, with few having intermediate CERAD levels. Among patients with a CERAD score of none, most had Braak stage 0 to II and Thal phase 0 to 2. Among patients with a CERAD score of frequent, most had Braak stage IV to VI and Thal phase 4 to 5.

### CSF Biomarkers by ADNC Class

**Figure 1:**
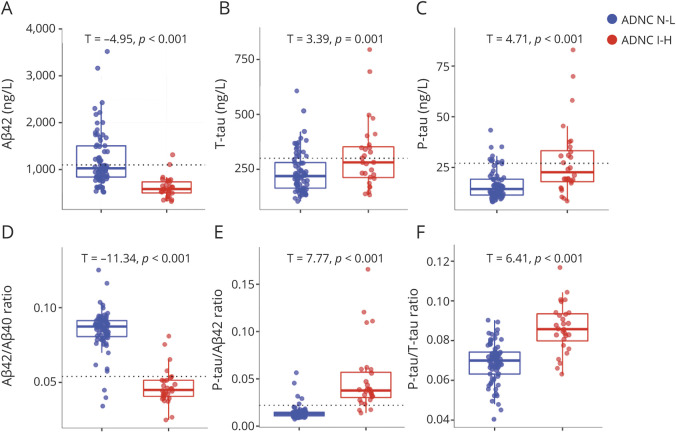
CSF Biomarkers by ADNC N-L vs I-H (A–F) Biomarkers are shown as unadjusted raw data in the groups of Alzheimer disease neuropathologic change (ADNC) none-low (N-L; blue) and intermediate-high (I-H; red). T values and *p* values are shown for group differences, adjusted for age, sex, and lag between lumbar puncture and death. Reference lines are shown for a priori cut points for β-amyloid (Aβ)_42_, total tau (T-tau), phosphorylated tau (P-tau), Aβ_42_/Aβ_40_, and P-tau/Aβ_42_, as defined previously.^43^ Aβ_40_ and neurofilament light biomarkers are shown in eFigure 3, links.lww.com/WNL/B777.

**Figure 2:**
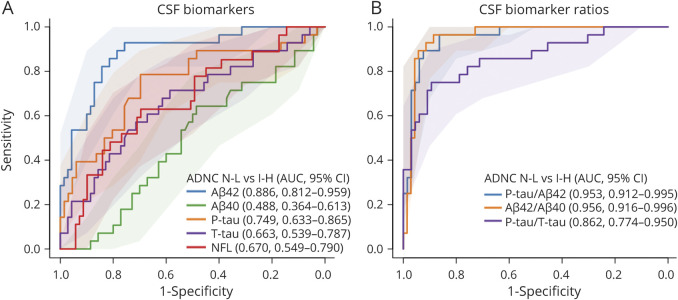
CSF Biomarkers for ADNC Classification Performance of logistic regression models for individual biomarkers (A) and biomarker ratios (B), to distinguish between Alzheimer disease neuropathologic change (ADNC) none-low (N-L) and ADNC intermediate-high (I-H). Legends show overall area under the receiver operating curve characteristics (AUC) with 95% CI from a bootstrap procedure. Aβ = β-amyloid; NFL = neurofilament light; P-tau = phosphorylated tau; T-tau = total tau.

We tested how well biomarkers differentiated between ADNC none-low (n = 70) vs ADNC intermediate-high (n = 28, ADNC data were missing in 3 patients due to missing CERAD score [n = 1] and/or Thal phase [n = 2] data). These comparisons were done regardless of other pathologies, but other pathologies were commonly reported as either primary or contributory in both ADNC none-low and ADNC intermediate-high (eTable 2, links.lww.com/WNL/B777). The ADNC intermediate-high group had significantly lower Aβ_42_ and Aβ_42_/Aβ_40_ and higher T-tau, P-tau, P-tau/Aβ_42_, and P-tau/T-tau compared to the ADNC none-low group (Figure 1). There were no differences in Aβ_40_ or NFL (note that all participants had cognitive impairment) (eFigure 3). We also tested the performance of the biomarkers in detecting ADNC intermediate-high in ROC analyses (Figure 2). The best individual biomarker was Aβ_42_ (AUC 0.886). Both P-tau/Aβ_42_ and Aβ_42_/Aβ_40_ ratios had very high performance (AUC 0.953–0.956) for separating ADNC none-low from ADNC intermediate-high. An internal 10-fold cross-validation of the estimates gave very similar results (eTable 3).

Time between LP and death was a possible confounder for associations between biomarkers and neuropathology (participant-level data on LP-to-death time are included in eFigure 4, links.lww.com/WNL/B777). In a sensitivity analysis, the individuals who were correctly classified by Aβ_42_ (concordance between predicted and observed ADNC class) had slightly longer lag time compared to those who were misclassified (*p* = 0.04, Mann-Whitney *U* test). There were no differences in lag time between correctly classified and misclassified groups for other biomarkers/ratios (*p* = 0.14–0.89).

An a priori defined cut point (derived in the external BioFINDER cohort, manuscript in preparation) for Aβ_42_/Aβ_40_ (<0.054) had high specificity for minimal AD pathology, defined as ADNC none-low (high Aβ_42_/Aβ_40_ was seen in 67 of 70 [96%] of patients with ADNC none-low), and high sensitivity for significant AD pathology, defined as ADNC intermediate-high (low Aβ_42_/Aβ_40_ was seen in 24 of 28 [86%] of patients with ADNC intermediate-high). Similar results were seen for an a priori defined cut point^43^ for P-tau/Aβ_42_ (>0.022) with high specificity (61 of 66 ADNC none-low, 92%) and high sensitivity (25 of 28 ADNC intermediate-high, 89%) for AD neuropathology. There were no differences in time from LP to death between correctly classified and misclassified individuals using the a priori cut points (*p* = 0.57–0.60). In sensitivity analyses, we tested the a priori cut points in subgroups of participants. In patients with a clinical diagnosis of AD (n = 15), both Aβ_42_/Aβ_40_ and P-tau/ Aβ_42_ were positive in 1 of the 2 patients with ADNC none-low. Aβ_42_/Aβ_40_ was positive in 12 of the 13 patients with ADNC intermediate-high, and P-tau/Aβ_42_ was positive in all 13 patients with ADNC intermediate-high. In patients with a non-AD diagnosis and ADNC data (n = 83, P-tau181 data were missing in 4 of these), Aβ_42_/Aβ_40_ was positive in 2 of 68 patients with ADNC none-low (97% specificity), and P-tau/Aβ_42_ was positive in 4 of 64 patients with ADNC none-low (94% specificity). Both Aβ_42_/Aβ_40_ and P-tau/Aβ_42_ were positive in 12 of 15 patients with ADNC intermediate-high (80% sensitivity).

### CSF Biomarkers by Thal Phase, Braak Stage, CERAD Score, and ADNC

**Figure 3:**
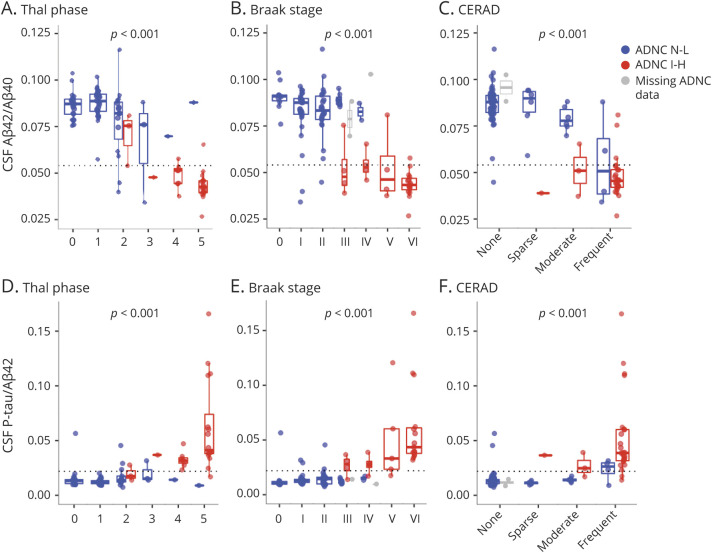
CSF Biomarkers by AD Neuropathologic Scores Biomarker ratios (β-amyloid [Aβ]_42_/Aβ_40_ and phosphorylated tau [P-tau]/Aβ_42_) are shown as unadjusted raw data by neuropathologic scores for spread of Aβ pathology (Thal phase 0–5), tau pathology (Braak stage 0–VI), and presence/frequency of neuritic plaques (Consortium to Establish a Registry for Alzheimer's Disease [CERAD] score). eFigure 5, links.lww.com/WNL/B777 gives Alzheimer disease (AD) neuropathologic change (ADNC) data. The *p* values are for the overall associations (from likelihood ratio tests of models with and without including the neuropathologic score as a predictor) between the neuropathologic scores and CSF biomarker levels, adjusted for age, sex, and time between lumbar puncture and death. A reference line is shown for an a priori cut point for Aβ_42_/Aβ_40_. Color coding refers to ADNC class (blue = ADNC none-low [N-L], red = ADNC intermediate-high [I-H], gray = missing ADNC data).

We next tested associations between biomarkers and Thal phase, Braak stage, CERAD score, and all levels of ADNC. Figure 3 shows data for Aβ_42_/Aβ_40_ and P-tau/Aβ_42_ (ADNC data are shown in eFigure 5, links.lww.com/WNL/B777). eFigure 6 (for Thal phase, Braak stage, and CERAD score) and eFigure 7 (for ADNC) show data for Aβ_42_, Aβ_40_, P-tau, T-tau, P-tau/T-tau, and NFL. In tests of overall associations (across all levels of the scores), lower levels of Aβ_42_ and Aβ_42_/Aβ_40_ and higher levels of P-tau, T-tau, P-tau/T-tau, and P-tau/Aβ_42_ were significantly associated with worse pathology. There were no significant associations for Aβ_40_ or NFL.

**Figure 4:**
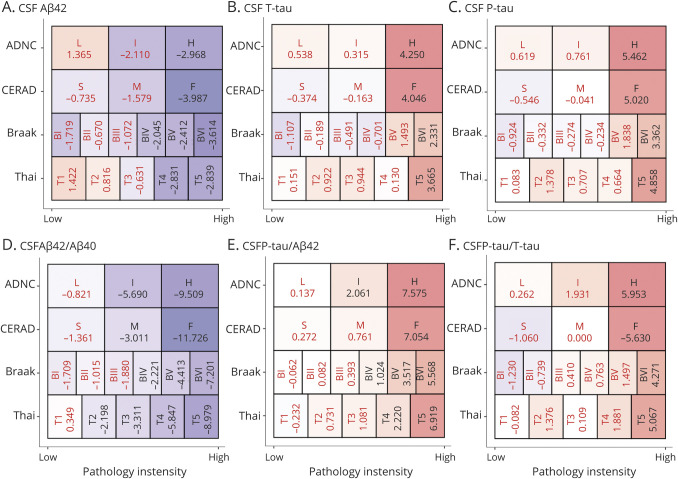
CSF Biomarker Changes at Different Levels of Pathology This figure shows how different biomarkers are altered at different levels of pathology compared to the lowest levels of respective pathology. Presented data are T statistics (black = significant at *p* < 0.05, red = nonsignificant). Box colors are related to the magnitude of the T statistics (red colors = positive, violet = negative). Data are presented for each biomarker (A–F) and the pathologic scores Thal phase (categories range from Thal phase 1–5), Braak stage (categories range from stage I–IV), Consortium to Establish a Registry for Alzheimer's Disease (CERAD) score (sparse [S], medium [M], frequent [F]), and Alzheimer disease neuropathologic change (ADNC) (low [L], intermediate [I], high [H]). For each score, biomarkers were compared between each category and the reference category (Thal phase 0, Braak stage 0, CERAD none, and ADNC no, respectively). The T statistics are from linear regression models, adjusted for age, sex, and time between lumbar puncture and death. For example, CSF β-amyloid (Aβ)_42_/Aβ_40_ (D) was significantly reduced at ADNC intermediate, CERAD moderate, Braak stage IV, and Thal phase 2 (and all greater levels of pathology). eFigure 8, links.lww.com/WNL/B777 gives results for Aβ_40_ and neurofilament light.

To test the sensitivity of biomarkers for lowest possible level of pathology, we compared biomarkers between every stage of the scores to the respective reference category (i.e., Thal phase 0, Braak stage 0, CERAD score of none, and ADNC none) (Figure 4). These models were corrected for age, sex, and time between LP and death. The first significant reductions in Aβ_42_ were seen in Thal phase 4, Braak stage IV, CERAD score of frequent, and ADNC intermediate. The first reductions in Aβ_42_/Aβ_40_ were seen in Thal phase 2, Braak stage IV, CERAD score of moderate, and ADNC intermediate. The first increases in P-tau/Aβ_42_ were seen in Thal phase 4, Braak stage V, CERAD score of frequent, and ADNC intermediate. These biomarker changes were always consistent at higher levels of pathology (e.g., Aβ_42_/Aβ_40_ remained changed at Thal phase 3–5). Significant increases in P-tau, T-tau, and P-tau/T-tau were seen only at the highest levels of pathology (Thal phase 5, Braak stage VI, CERAD score of frequent, and ADNC high). For Aβ_40_, there was an increase at Thal phase 2, but this was not seen at greater Thal phases, and there were no significant changes in NFL (eFigure 8, links.lww.com/WNL/B777).

We considered the possibility that associations between biomarkers and pathology were driven partly by group differences between AD and FTLD. In a sensitivity analysis, we adjusted all models for AD as a primary pathology. This attenuated associations for Aβ_42_, P-tau, T-tau, and the P-tau/T-tau ratio but associations for the Aβ_42_/Aβ_40_ and P-tau/Aβ_42_ ratios with neuropathology were robust (eTable 4, links.lww.com/WNL/B777). The lowest pathology levels with significant biomarker changes were slightly attenuated (eFigure 9). Aβ_42_/Aβ_40_ was significantly reduced from Thal phase 2, Braak stage V, CERAD score of moderate, and ADNC intermediate, while P-tau/Aβ_42_ was significantly increased from Thal phase 5, Braak stage V, and CERAD score of frequent and ADNC high.

Although we included time from LP to death as a covariate in the models, we considered the possibility that differences in lag time could still affect the findings. In another sensitivity analysis, we therefore excluded individuals with more than the median lag time (>2.67 years). This did not alter the overall associations between biomarkers and pathology (eTable 4, links.lww.com/WNL/B777). There was no indication that the biomarkers had greater sensitivity for lower grade of pathology in the subset with short time from LP to death (eFigure 10). We also evaluated the interaction between pathology and time from LP to death to predict CSF biomarkers, which was generally not significant (eFigure 11).

We also considered education and level of cognitive impairment as possible confounders (although they were not significantly associated with the biomarkers). We refit the models when also adjusting for years of education and baseline Mini-Mental State Examination score. This did not affect associations between biomarkers and pathology (eTable 4, links.lww.com/WNL/B777).

### CSF Biomarkers by Combinations of Thal Phase, Braak Stage, and CERAD Score

To clarify whether biomarkers depended on 1 or several of Thal phase, Braak stage, and CERAD, we compared models with different sets of predictors of CSF biomarkers: a basic model using only age, sex, and time between LP and death (these were also included as covariates in all models below); only Thal phase; only Braak stage; only CERAD score; and combinations of 2 or 3 pathologic features. Models were compared in terms of AIC and *R*^2^ (eTable 5, links.lww.com/WNL/B777). For Aβ_40_ and NFL, no pathology model was better than the basic model. The Thal phase–only model was preferred for Aβ_42_ (*R*^2^ = 0.21, ΔAIC = −18.1 compared to the basic model). The Thal phase and CERAD model was preferred for Aβ_42_/Aβ_40_ and P-tau/Aβ_42_ (*R*^2^ = 0.33, ΔAIC = −27.9; *R*^2^ = 0.24, ΔAIC = −16.3, respectively). The Braak stage–only model was preferred for P-tau/T-tau (*R*^2^ = 0.41, ΔAIC = −34.2).

### CSF Biomarkers by Primary Neuropathologic Diagnosis

**Figure 5:**
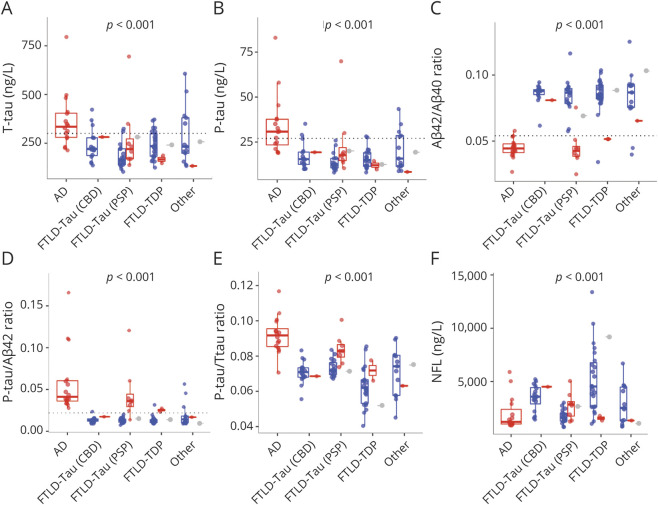
CSF Biomarkers by Primary Pathologic Diagnosis Biomarkers (A–F) are shown as unadjusted raw data by primary neuropathologic diagnosis. The *p* values are shown for overall significance of neuropathologic diagnosis, adjusted for age, sex, and time between lumbar puncture and death. eFigure 12, links.lww.com/WNL/B777 gives results for β-amyloid (Aβ)_40_ and Aβ_42_. eTable 6 gives pairwise comparisons between different diagnoses. Reference lines are shown for a priori cut points for total tau (T-tau), phosphorylated tau (P-tau181), Aβ_42_/Aβ_40_, and P-tau/Aβ_42_, as defined previously.^43^ Color coding refers to Alzheimer disease neuropathologic change (ADNC) class (blue = ADNC none-low, red = ADNC intermediate-high, gray = missing ADNC data). AD = Alzheimer disease; CBD = corticobasal degeneration; FTLD = frontotemporal lobar degeneration; PSP = progressive supranuclear palsy; TDP = TAR DNA binding protein 43.

We tested associations between primary neuropathologic diagnosis and biomarkers. All biomarkers except Aβ_40_ differed across groups (Figure 5 and eFigure 12, links.lww.com/WNL/B777). We also compared biomarkers pairwise between groups (adjusted for multiple comparisons with the Bonferroni method for 10 comparisons). These comparisons are summarized in eTable 6. AD had significantly lower Aβ_42_/Aβ_40_ ratio and higher P-tau, P-tau/Aβ_42_, and P-tau/T-tau than all other groups. AD also had lower Aβ_42_ than FTLD-tau (CBD), FTLD-TDP, and other and higher T-tau than FTLD-tau (CBD), FTLD-tau (PSP), and FTLD-TDP. Patients with FTLD-tau (PSP) had higher P-tau/T-tau than patients with FTLD-TDP. Patients with FTLD-TDP had higher NFL than those with AD, FTLD-tau (PSP), and other.

Twelve patients without AD (1 FTLD-tau [CBD], 8 FTLD-tau [PSP], 2 FTLD-TDP, and 1 other) had intermediate-high ADNC (shown in red in Figure 5). When excluding these patients and repeating the pairwise comparisons (eTable 6), we find that patients with FTLD-TDP had higher NFL than FTLD-tau (CBD), in addition to the other groups. The difference in P-tau/T-tau between FTLD-tau (PSP) and FTLD-TDP was attenuated and no longer significant after correction for multiple comparisons (uncorrected *p* = 0.0065).

### A Priori Cut Points for CSF Biomarkers and Primary Neuropathologic Diagnosis

We evaluated a priori cut points for Aβ_42_, P-tau, T-tau, Aβ_42_/Aβ_40_, and P-tau/Aβ_42_ for primary neuropathologic diagnosis in terms of sensitivity for AD and specificity for non-AD. Aβ_42_, Aβ_42_/Aβ_40_, and P-tau/Aβ_42_ had 94% to 100% sensitivity for AD (Table 1). Overall, for non-AD, Aβ_42_ had 38% specificity, T-tau had 79% specificity, P-tau and Aβ_42_/Aβ_40_ had 86% specificity, and P-tau/Aβ_42_ had 83% specificity (Table 1 shows details by individual variants).

For patients without AD, we tested whether biomarker status (positive or negative) varied with comorbid ADNC (none-low vs intermediate-high). These analyses showed that among patients with diseases other than AD as primary pathology (non-AD), positive Aβ_42_/Aβ_40_ or P-tau/Aβ_42_ biomarker ratios (but not individual biomarkers) were significantly associated with AD copathology (eTable 7, links.lww.com/WNL/B777).

### CSF Biomarkers by Primary and Contributory Neuropathologic Diagnosis

**Figure 6:**
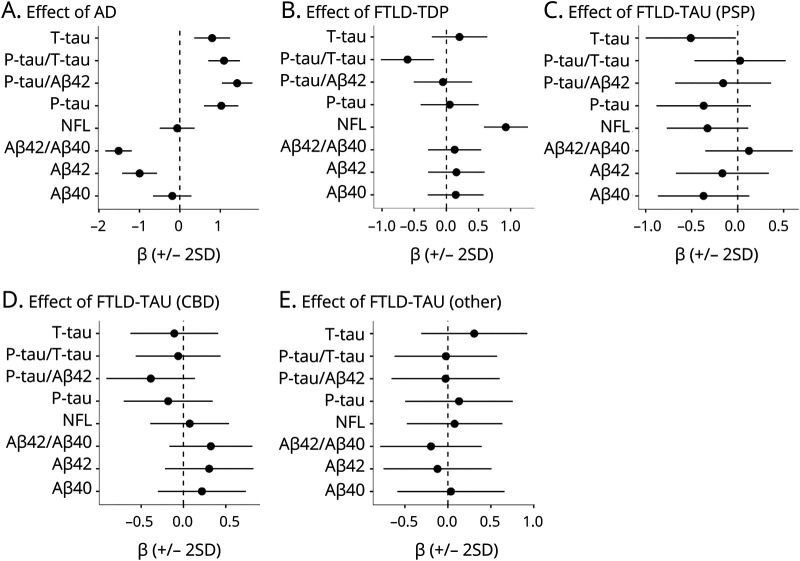
CSF Biomarkers vs Primary and Contributory Neuropathology Diagnoses Effects are plotted for each neuropathologic class (A–E) from linear regression models adjusted age, sex, and time between lumbar puncture and death. Coefficients represent the average difference in biomarkers between patients who were positive for a neuropathology (e.g., AD in panel A, also if only present as a copathology) compared to the remaining patients. AD = Alzheimer disease; CBD = corticobasal degeneration; FTLD = frontotemporal lobar degeneration; NFL = neurofilament light; P-tau = phosphorylated tau; PSP = progressive supranuclear palsy; T-tau = total tau; TDP = TAR DNA binding protein 43.

Because the designation of primary neuropathologic diagnosis was not blinded to the clinical syndrome, we also classified each patient as positive or negative for AD (n = 28 positive), FTLD-TDP (n = 32 positive), FTLD-tau (PSP) (n = 26 positive), FTLD-tau (CBD) (n = 19 positive), or FTLD-tau (other) (n = 12 positive) independently of whether the neuropathologic diagnosis was called primary or contributory (meaning that a patient could be positive for >1 of these). Associations were tested between these classifications and biomarkers (Figure 6). AD neuropathologic diagnosis was associated with significantly altered levels (in the expected directions) of all biomarkers except Aβ_40_ and NFL. FTLD-TDP pathology was associated with reduced P-tau/T-tau and elevated NFL. FTLD-tau (PSP) was associated with reduced T-tau.

Note that these analyses were not adjusted for copathologies. However, we also performed these tests for the FTLD diagnoses after removing patients with intermediate-high ADNC (eFigure 13, links.lww.com/WNL/B777), leaving 24 with FTLD-TDP, 17 with FTLD-tau (PSP), 16 with FTLD-tau (CBD), and 9 with FTLD-tau (other). FTLD-TDP pathology remained associated with reduced P-tau/T-tau and elevated NFL, and FTLD-tau (PSP) remained associated with reduced T-tau. FTLD-tau (other) was associated with elevated P-tau/Aβ_42_, T-tau, and P-tau.

### Genetic Variants, CSF Biomarkers, and Neuropathology

Data on genetic variants were available for *APOE* ε4 (positive n = 26, negative n = 74, Table 1), *C9orf72* variations (positive n = 8 [all FLTD-TDP], negative n = 92), *GRN* variations (positive n = 5 [1 AD, 4 FTLD-TDP], negative n = 95), and *MAPT* variations (positive n = 2 [both classified as other, with FTD-PPA syndromes], negative n = 96).

*APOE* ε4 was associated with lower Aβ_42_ (*p* = 0.028) and Aβ_42_/Aβ_40_ (β = −0.019, *p* < 0.001) and higher P-tau/Aβ_42_ (β = 0.017, *p* = 0.0056). After adjustment for Thal phase, Braak stage, and CERAD score (used together), these associations were lost (Aβ_42_ *p* = 0.91, Aβ_42_/Aβ_40_ *p* = 0.91, P-tau/Aβ_42_ *p* = 0.87), supporting that *APOE* variants affect CSF biomarker levels indirectly through neuropathology.

Among patients with FTLD primary pathologies, there were no associations between *C9orf72* variations (n = 8 positive, n = 62 negative) and biomarker levels. The other variations had too few cases for meaningful analyses.

### Classification of Evidence

This study provides Class II evidence that distinct CSF biomarker patterns, including for P-tau, T-tau, Aβ_42_, Aβ_40_, and NFL, are associated with AD and FTLD neuropathology.

## Discussion

This clinicopathologic CSF biomarker study in patients with AD and FTLD, which included Aβ_42_/Aβ_40_, P-tau, and P-tau/Aβ_42_ measured with fully automated Elecsys assays and NFL measured with an ultrasensitive Simoa assay, found that specific biomarkers were strongly correlated with AD, including when AD was present as copathology in patients with another primary pathology. The Aβ_42_/Aβ_40_ and P-tau/Aβ_42_ ratios had very high overall accuracy (AUC 0.95–0.96) to detect significant AD pathology, defined as intermediate-high ADNC. Although most of the biomarker changes were associated with quite advanced neuropathology, group-level changes in CSF Aβ_42_/Aβ_40_ appeared already at Thal phase 2, supporting that selected CSF biomarkers may begin to be influenced at low levels of AD pathology. However, the biomarkers did not have sensitivity for single-participant–level prediction at very early stages of Aβ or tau pathologies in this study. Another key finding was that autopsy-confirmed FTLD variants displayed group-level biomarker patterns, including reduced CSF T-tau in FTLD-tau (PSP) and reduced CSF P-tau/T-tau and increased CSF NFL in FTLD-TDP. Taken together, these findings support that CSF biomarkers can be used to identify underlying neuropathologic AD changes, and they are also differentially expressed in different FTLD pathologies. This is one of few studies that combine careful characterization of neuropathologic features with the use of only recently available modern assays for CSF AD biomarkers and NFL. The findings support the use of these biomarkers to characterize the underlying neuropathologic changes in patients with AD and FTLD.

There were important findings for several of the individual biomarkers and ratios. In relation to Aβ pathology, we noted that Aβ_42_/Aβ_40_ levels started to change already at Thal phase 2, which may be comparable to what has been described previously for Aβ PET in an overlapping cohort.^44^ Previous studies directly comparing CSF Aβ and Aβ PET biomarkers have suggested that CSF Aβ measures may be altered before Aβ PET in response to altered Aβ metabolism.^45^ We note that we did not generally find that CSF biomarkers were altered in very early stages of neuropathology. This was especially true for tau accumulation, for which no biomarker was significantly altered before Braak stage IV. Although this study did not directly compare CSF and PET measures with neuropathology, our findings suggest that the CSF biomarkers studied here were not significantly altered before changes may also be detected by PET imaging. However, a possible caveat for CSF studies of very early changes with regard to neuropathology is the delay between LP and death, which may obscure the exact relationships between subtle biomarker changes and the first neuropathologic changes. Another caveat is that truly quantitative data on Aβ pathology, rather than the semiquantitative data used here, may be necessary to find subtle differences in when CSF and PET biomarkers start to change with respect to pathology. Furthermore, studies that find evidence of early CSF changes before changes in PET measures have typically seen such discrepancy in people without cognitive impairment or only mild cognitive impairment, which is different from the current study population, in which most research participants had dementia. The findings for the P-tau/Aβ_42_ ratio were similar to those for Aβ_42_/Aβ_40_, with high diagnostic accuracy for AD neuropathology. We found pronounced changes in P-tau/Aβ_42_ also when AD was a copathology in FTLD. Theoretically, a high P-tau/Aβ_42_ ratio may reflect a combination of Aβ pathology and AD-specific axonal degeneration with tangle material. The ratio correlated with all of Thal, Braak, and CERAD scores, although significant changes seemed to appear later than for Aβ_42_/Aβ_40_ (Thal phase 3 instead of Thal phase 2, Braak stage V instead of Braak stage IV).

The individual CSF Aβ_42_ measure was reduced in patients with AD (as expected) but also in several patients with FTLD, as described before in FTLD cohorts.^17^ We found that reductions of Aβ_42_ were seen mainly in patients with FTLD with concomitant AD pathology (defined as intermediate-high ADNC), but several patients with FTLD had low CSF Aβ_42_ levels despite having none-low ADNC grade (Figure 5B). In contrast, the CSF Aβ_42_/Aβ_40_ ratio was very rarely reduced in patients with FTLD without concomitant Aβ pathology (Figure 5E). This supports that CSF Aβ_42_/Aβ_40_ can be used a tool to detect concomitant AD pathology in FTLD and illustrates the importance of correcting CSF Aβ_42_ levels for a shorter Aβ isoform such as Aβ_40_ or Aβ_38_ to detect underlying Aβ pathology. Hypothetically, other factors beyond Aβ pathology may have contributed to the reduced levels of Aβ_42_ (without affecting the Aβ ratio) in patients with FTLD, including white matter disease, neuroinflammation, and neuronal loss leading to reduced overall Aβ release.

CSF T-tau, which is nonspecifically elevated in several conditions with axonal injury, generally did not reach AD levels in the patients with FTLD in this study. Low levels of T-tau were seen in PSP, which is in agreement with previous studies based on clinical diagnosis.^13,17^ We have no clear explanation for the low tau levels in PSP. Hypothetically, it could be related to involvement of cortical vs subcortical structures (cortical abnormalities may presumably be more readily detected as CSF changes compared to subcortical abnormalities, which dominate in PSP). The low levels of T-tau in PSP may also be related to disease-specific differences in tau fragment confirmations or in neuronal releases of 3R vs 4R tau.^13^ Hypothetically, CSF tau levels could also be reduced due to sequestration of tau in tangles in tauopathies. We note that the reduced tau levels seem to be more specific for PSP than for CBD, which is interesting given the similarities between these conditions neuropathologically. P-tau, which has been associated with buildup of AD-specific tau aggregates, did not clearly differentiate between Aβ plaques and neurofibrillary tangles in this cohort because associations were seen between high P-tau and neuropathologic measures both of tau and Aβ pathology. Soluble P-tau may be increased in response to Aβ buildup as a first indicator of altered tau metabolism in individuals who are positive on Aβ PET but negative on tau PET.^46^ However, we did not find that P-tau was altered in response to early Aβ pathology, which may be expected given previous PET studies. The relationship between soluble P-tau and buildup of aggregated Aβ and tau fibrils is complex and warrants further studies. We focused on P-tau181, but other variants of P-tau (including P-tau217) may also be interesting to study in relation to neuropathology. P-tau levels were generally low in FTLD. Combining P-tau and T-tau in a ratio may integrate information about phosphorylation and release of tau proteins. Theoretically, a low P-tau/T-tau ratio may reflect axonal degeneration without AD-type degeneration because T-tau (but not P-tau) is increased nonspecifically due to axonal degeneration. Among the different AD pathology features, the P-tau/T-tau ratio was most closely associated with Braak stage (rather than Thal phase and CERAD score, eTable 6, links.lww.com/WNL/B777). The ratio increased separation between some of the groups compared to the individual tau measures. In particular, P-tau/T-tau was reduced in individuals with FTLD-TDP, which is in line with previous findings based on clinical, neuropathologic, and genetic evidence.^17,18^ The FTLD-TDP group was further characterized by increased NFL, which is also in line with previous literature.^47^ One biomarker-pathology study in AD and FTLD that compared CSF T-tau and NFL in a panel of biomarkers found that NFL improved diagnostic accuracy, further supporting that NFL can provide important information.^48^

One strength of this study is that we had neuropathologic data in all patients, combined with CSF samples, and the sample size was relatively large for an antemortem biomarker vs neuropathology study. A limitation is that the sample size was relatively small for some of the subgroups. A larger population will make it easier to fully disentangle associations between biomarkers and specific pathologic features. Additional weaknesses include the lack of a control population for normal aging, as well as the lack of groups with other dementias such as Lewy body dementia or vascular dementia. Future studies may include these groups to increase the generalizability of the findings. The time interval between LP and death may obscure relationships between CSF biomarkers and neuropathologic features, although we adjusted for this.

CSF Aβ_42_/Aβ_40_, T-tau, P-tau, P-tau/Aβ_42_, and NFL were related to underlying neuropathologic changes of AD and FTLD variants. An AD-like biomarker profile supports AD comorbidity in patients with FTLD. In contrast, a non–AD-like biomarker profile, with reduced P-tau/T-tau and increased NFL, may support FTLD-TDP rather than FTLD-tau lesions in the brain. Low T-tau may support FTLD-tau (PSP) pathology rather than other FTLD pathologies. Further work is needed to assess how these findings can be translated to blood-based biomarkers, which have shown promising results with high diagnostic accuracy for AD and underlying pathologic changes.^49,50^ Further work is also needed to test how these biomarker profiles perform at the single-participant level, and the findings need to be replicated in an independent sample.

## Glossary

Aβ: β-amyloid
AD: Alzheimer disease
ADNC: AD neuropathologic change
AIC: Akaike information criterion
AUC: area under the ROC curve
bvFTD: behavioral variant FTD
CBD: corticobasal degeneration
CBS: corticobasal syndrome
CERAD: Consortium to Establish a Registry for Alzheimer's Disease
FTD: frontotemporal dementia
FTLD: frontotemporal lobar degeneration
LP: lumbar puncture
NFL: neurofilament light chain
P-tau: phosphorylated tau
PSP: progressive supranuclear palsy
ROC: receiver operating characteristics
T-tau: total tau
TDP-43: TAR DNA-binding protein 43
UCSF: University of California San Francisco
